# The Mitochondrial Na^+^/Ca^2+^ Exchanger Upregulates Glucose Dependent Ca^2+^ Signalling Linked to Insulin Secretion

**DOI:** 10.1371/journal.pone.0046649

**Published:** 2012-10-08

**Authors:** Iulia I. Nita, Michal Hershfinkel, Daniel Fishman, Eyal Ozeri, Guy A. Rutter, Stefano L. Sensi, Daniel Khananshvili, Eli C. Lewis, Israel Sekler

**Affiliations:** 1 Department of Physiology, Faculty of Health Sciences, Ben-Gurion University of the Negev, Beer-Sheva, Israel; 2 Department of Morphology, Faculty of Health Sciences, Ben-Gurion University of the Negev, Beer-Sheva, Israel; 3 Department of Clinical Biochemistry, Faculty of Health Sciences, Ben-Gurion University of the Negev, Beer-Sheva, Israel; 4 Section of Cell Biology, Division of Diabetes, Endocrinology and Metabolism, Department of Medicine, Faculty of Medicine, Imperial College London, London, United Kingdom; 5 Department of Neuroscience and Imaging, University G. d'Annunzio, Chieti-Pescara, Italy; 6 Molecular Neurology Unit, Center of Excellence on Aging, University G. d'Annunzio, Chieti-Pescara, Italy; 7 Department of Physiology and Pharmacology, Sackler School of Medicine, Tel Aviv University, Ramat-Aviv, Israel; Université Joseph Fourier, France

## Abstract

Mitochondria mediate dual metabolic and Ca^2+^ shuttling activities. While the former is required for Ca^2+^ signalling linked to insulin secretion, the role of the latter in β cell function has not been well understood, primarily because the molecular identity of the mitochondrial Ca^2+^ transporters were elusive and the selectivity of their inhibitors was questionable. This study focuses on NCLX, the recently discovered mitochondrial Na^+^/Ca^2+^ exchanger that is linked to Ca^2+^ signalling in MIN6 and primary β cells. Suppression either of NCLX expression, using a siRNA construct (siNCLX) or of its activity, by a dominant negative construct (dnNCLX), enhanced mitochondrial Ca^2+^ influx and blocked efflux induced by glucose or by cell depolarization. In addition, NCLX regulated basal, but not glucose-dependent changes, in metabolic rate, mitochondrial membrane potential and mitochondrial resting Ca^2+^. Importantly, NCLX controlled the rate and amplitude of cytosolic Ca^2+^ changes induced by depolarization or high glucose, indicating that NCLX is a critical and rate limiting component in the cross talk between mitochondrial and plasma membrane Ca^2+^ signalling. Finally, knockdown of NCLX expression was followed by a delay in glucose-dependent insulin secretion. These findings suggest that the mitochondrial Na^+^/Ca^2+^ exchanger, NCLX, shapes glucose-dependent mitochondrial and cytosolic Ca^2+^ signals thereby regulating the temporal pattern of insulin secretion in β cells.

## Introduction

Cross talk between the plasma membrane and mitochondria is essential for mediating glucose-dependent insulin secretion. Cellular uptake and metabolism of glucose by pancreatic β cells stimulates ATP production [1], [2]. The subsequent rise of cytosolic ATP initiates cellular depolarization by inhibition of ATP-sensitive K^+^ channels, opens L-type Ca^2+^ channels (LTCC), induces a rise in cytosolic Ca^2+^ and leads to insulin secretion [3].

Mitochondria however, are also a major hub for cellular Ca^2+^ transport that is powered by their steep membrane potential. Cytosolic increase in Ca^2+^ is followed by Ca^2+^ influx into the mitochondria via the mitochondrial uniporter recently shown to be linked to MCU (mitochondrial Ca^2+^ uniporter) [4], [5]. In the mitochondrial matrix, Ca^2+^ is buffered by calcium phosphate and subsequently extruded by an electrogenic 3Na^+^/Ca^2+^ exchanger that uses the mitochondrial membrane potential and Na^+^ gradient to pump Ca^2+^ out of the mitochondria back into the cytoplasm [6]. The mitochondrial Ca^2+^ shuttling has several roles: first, Ca^2+^ activates at least three key intra-mitochondrial dehydrogenases [7], [8] and hence, serves as a key regulator of the rate of ATP synthesis. Second, the mitochondria are a major and highly dynamic Ca^2+^ store and the Ca^2+^ efflux by the mitochondrial exchanger can control the amplitude and duration of cytosolic Ca^2+^ transients, for example, in neurons [9] and chromaffin cells [10]. Active influx and Na^+^ dependent efflux of Ca^2+^ has been described also in isolated mitochondria from β cells [11]. However, the role of mitochondrial Ca^2+^ shuttling, and in particular, the mitochondrial Na^+^/Ca^2+^ exchanger in glucose dependent Ca^2+^ signalling in β cells are not well understood. A major complication is that the molecular identity of the mitochondrial Na^+^/Ca^2+^ exchanger has been elusive until recently and the concern that the inhibitor of the exchanger, CGP-37157 may interact with other major Ca^2+^ transport pathways in β cells. For example, CGP-37157 was suggested to trigger mitochondrial Ca^2+^ rise by blocking the exchanger thereby leading to enhanced mitochondrial oxidative metabolism and insulin secretion [12]. However a subsequent study [13], suggested that CGP-37157 also affects cytosolic Ca^2+^ signals by blocking the LTCC in β cells [14]. Other studies further indicated that CGP-37157, like other benzothiazepin compounds, may also modulate the activity of other major Ca^2+^ transporters among them: sarcoma(-endo) plasmic reticulum Ca^2+^-ATPase, SERCA, and ryanodine receptors, RyR [15].

We and subsequently others, found that the Na^+^/Ca^2+^ exchanger super family member NCLX is localized in the mitochondria where it mediates Ca^2+^ efflux and is therefore likely the mitochondrial Na^+^/Ca^2+^ exchanger [16], [17]. We further showed that expression of NCLX is effectively attenuated by small interfering RNA construct and its activity blocked by a catalytic inactive NCLX (dnNCLX) that induce a strong dominant negative effect on the endogenous exchanger activity [16]. Using these molecular tools, siNCLX and dnNCLX, derived from the cloning of NCLX, we sought to determine in the present study the general role of NCLX in shaping cytosolic and mitochondrial Ca^2+^ signalling linked to insulin secretion. Our results indicate that NCLX is not only critical for mitochondrial Ca^2+^ efflux but also affects cytoplasmic Ca^2+^ responses. A major conclusion of this study is that mitochondrial Ca^2+^ shuttling, catalyzed by NCLX, plays a dominant role in shaping glucose-dependent cytosolic Ca^2+^ transients and thereby regulates the temporal pattern of insulin secretion.

## Methods

### Mice

Six-eight week old female DBA/2J mice were purchased from Jackson laboratories, Bar Harbor, ME, USA. Mice were kept in a pathogen-free environment at the Ben-Gurion University of the Negev Research Animal Facility and were cared for according to the Ben-Gurion University of the Negev Care and Use of Animals committee guidelines.

### Islet isolation

Animals were anesthetized prior to islet harvest by standard ketamine/xylazine and islets were isolated by collagenase digestion [18]. Briefly, digested pancreata were filtered through 1000 µm and 500 µm sieves and then hand-picked under a stereoscope as previously described [18].

### Cell culture and transfection

Isolated islets were cultured in RPMI 1640 (Beit Haemek, 01-100 1A, Israel) for 2–3 days and MIN6 cells in DMEM (Beit Haemek, 01-055-1A). Both media were supplemented with 10% fetal calf serum (Beit Haemek, 04-001-1A), 1% Pencillin/Streptomycin (Beit Haemek, 02-020-1B), 1% L-Glutamine (Beit Haemek, 03-020-1B), 5 mM Glucose (Gerbu, 2028). Islets were hand-picked under a stereoscope and dispersed into single cells by Trypsin-EDTA (Beit Haemek, 03-053-1B). Dispersed primary islet cells and MIN6 cells were seeded onto coverslips for imaging experiments [19]. Pancreatic β cells were identified in primary islets using anti-human/bovine/mouse insulin monoclonal antibody. Immunohistochemical analysis of insulin were performed and showed that more than 90% of cells in the culture were β cells as previously described [20].

Pancreatic primary β cells and MIN6 cells cultured on glass coverslips were transfected with siNCLX vs. siControl using DharmaFECT siRNA Transfection Reagents (Dharmacon, T-2004). siRNA NCLX or siControl was diluted in DharmaFECT siRNA transfection reagent, incubated approximately 20 min at room temperature and then added in the antibiotics free media. The efficiency of transfection was assessed by visualizing Dharmacon siGLO Red (Dharmacon, D-001630-02-05) transfection particles according to the protocol provided by manufacturer. The transfection efficiency, for siNCLX delivery as determined by siGlo fluorescent marker was high, ∼80%. Pancreatic primary β cells and MIN6 cells were co-transfected with a dominant negative active form of NCLX [16], dnNCLX vs. pcDNA and EYFP using Lipofectamine 2000 (Invitrogen, 11668-019). The constructs dnNCLX or pcDNA, were diluted in Lipofectamine 2000, gently mixed and added to plates of cells containing antibiotics free media. Successfully transfected cells were identified by monitoring EYFP fluorescence. Note that EYFP fluorescence does not interfere with monitoring Fura-2 AM (Teflabs, 0102) fluorescence [21]. Typically, 10% of the pancreatic primary β cells were successfully transfected and used for analysing the effect of dnNCLX consistent with the relatively low transfection efficacy of β primary and cell lines [22]. Because the imaging system allows the selection of single cells sufficient number of cell could be obtained despite the relatively low transfection efficiency. Fluorescent ion measurements were conducted 48 h after transfection. Cell viability was determined by monitoring Trypan Blue staining according to a protocol described elsewhere [23].

### Fluorescent Ca^2+^ imaging

The imaging system consisted of an Axiovert 100 inverted microscope (Zeiss), Polychrome V monochromator (TILL Photonics, Planegg, Germany) and a SensiCam cooled charge-coupled device (PCO, Kelheim, Germany). Fluorescent images were acquired with Imaging WorkBench 4.0 (Axon Instruments, Foster City, CA). Ca^2+^ imaging was performed in pancreatic primary β cells and MIN6 cells attached onto coverslips and superfused with Ringer solution containing (in mM): 126 NaCl (Frutarom, 2355534700067), 5.4 KCl (Sigma, P9333), 0.8 MgCl_2_ (Gerbu, 1722), 20 HEPES (Amresco, 0511), 1.8 CaCl_2_ (Sigma, C1016), 15 Glucose (Gerbu, 2028), pH adjusted to 7.4 with NaOH (Sigma, S8045) or with high K^+^ (50 mM) Ringer solution when indicated. In glucose dependent experiments the pancreatic primary β cells or MIN6 cells were pre-washed for 30 min with low glucose (3 mM) Ringer solution followed by high glucose (20 mM) Ringer solution.

Pancreatic primary β cells and MIN6 cells were loaded with Fura 2AM, excited with 340/380 nm wavelength light and imaged using a 510 nm long pass filter [24]. Mitochondrial Ca^2+^ measurements were performed in pancreatic primary β cells infected with lenti-pericam and in MIN6 cells expressing ratiometric mitochondrial pericam, which is targeted solely to the inner membrane of mitochondria [25], [26]. The mitochondrial pericam fluorescence in both pancreatic primary islet cells and MIN6 cells was acquired at 430 nm excitation and 550 nm emission as previously described [25].

### Fluorescent imaging of metabolic rate and mitochondrial membrane potential

Mitochondrial membrane potential was monitored in MIN6 cells loaded and superfused with TMRM (Invitrogen, T-668), excited at 545 nm and imaged with a 570 nm emission filter as previously described [27]. NAD(P)H intrinsic fluorescence in pancreatic primary β cells was fluorescently monitored (360 nm excitation and 440 nm emission) in an inverted microscope (see *Fluorescent Ca^2+^ imaging*), as previously reported [28] and calibrated by superfusing the cell with a Ringer solution containing 5 µM FCCP, protonophore carbonyl cyanide 4-(trifluoromethoxy)phenylhydrazone (Ascent Laboratories, Asc-081) at the end of the experiments [29].

### Cell fractionation and immunoblot analysis

Cell fractionation was performed as previously described [30]. Briefly, MIN6 cells were homogenized using a glass Douncer homogenizer with ice cold isolation buffer containing (in mM): 225 Manitol (Sigma, M4125), 75 Sucrose (Sigma, S7903), 0.5 EDTA (Sigma, E5134), 10 HEPES (Amresco, 0511), pH adjusted to 7.4 with KOH (Sigma, P5958). The lysates were centrifuged at 1500×g for 5 min at 4°C, supernatant was re-suspended in 200 µl of the same buffer and re-centrifuged for 4 min at 12,000×g at 4°C. The pellet, containing the mitochondrial fraction was then diluted with 100 µl cold isolation buffer and used immediately for immunoblot analysis. Protein concentration was determined by a modified Lowry procedure, according to manufacturer's protocol (Bio-Rad). Equal amounts of protein (20 µg) from total fraction and pure mitochondria samples were resolved by SDS-PAGE and transferred onto nitrocellulose membranes. Immunoblot analysis was performed as described previously using antibody generated against NCLX (1∶1000) and against VDAC (1∶1000), diluted into 5% milk (Fluka, 70166) in Tris-buffered saline solution with Tween 20 (Sigma, P1379) [31].

### Real time PCR Analysis

RNA was isolated from pancreatic primary islet cells transfected with either siControl ( *AACGGCCACUCAACUGUCU* ) or siNCLX ( *AACGCGCAUCCAACUGUCU* ) using TRIZol reagent (Invitrogen, 15596-026) following the manufacturer's instructions. The pancreatic primary islets were homogenized in TRIZol, followed by phase separation with chloroform and centrifuged at 12000×g for 15 min at 2°C. RNA was then precipitated from the aqueous phase by mixing with an equal volume of isopropyl alcohol and centrifuged at 12000×g for 10 min at 2°C. Finally, the RNA pellet was dried and dissolved in RNA-ase free water. The cDNA was generated using First Strand cDNA Synthesis Kit (Fermentas, K1611). Thermal cycling (40 cycles) conditions were 50°C for 15 min, followed by 95°C for 15 min, 95°C for 15 sec and 60°C for 60 sec. As reference gene, we employed glyceraldehyde 3-phosphate dehydrogenase (GAPDH). PCR reactions were conducted using a SDS7500 Real Time PCR machine and TaqMan probes (ThermoScientific).

### Cellular ATP content

Pancreatic primary islets cells were kept in low glucose (3 mM) Ringer solution for 30 min that was then replaced with high glucose (20 mM) containing Ringer solution. Cells were lysed at the indicated time intervals and ATP content was determined using the luciferin/luciferase luminescence assay with Bioluminescent Cell Titer-Glo Assay Kit (Promega, G7570) according to the manufacturer's instructions.

### Insulin secretion

Insulin secretion was monitored using a commercial ELISA kit (Mercordia, 10-1247-01). Pancreatic islet cells were incubated for 30 min in low glucose (3 mM) Ringer solution and then stimulated with high glucose (20 mM) Ringer solution. Aliquots were collected at the indicated time intervals and the amount of insulin secreted from pancreatic primary islet cells was determined according to the manufacturer's protocol (Mercordia mouse insulin ELISA kit).

### Statistical analysis

The traces of all fluorescent imaging experiments were plotted using KaleidaGraph 4.0. Influx of Ca^2+^ into the cytosol or mitochondria consistently began after stimulation of pancreatic β cells with High K^+^, ATP or high glucose. Similarly, Ca^2+^ efflux out of the mitochondria followed the influx phase as previously described [16]. The fluorescent ratio signals were normalized to the average signal obtained at the beginning of the measurements. The influx and efflux rates were calculated as slope of the linear fit of the fluorescence change during 30 seconds following the given stimulation (i.e. High K^+^, ATP or high glucose) for Ca^2+^ influx or Ca^2+^ efflux as previously described [16]. Peak amplitude was defined according to established protocols [32], comparing the maximal peak height of the signal to the background fluorescence. Changes in rate of the fluorescence Ca^2+^ response or their amplitude (ΔCa^2+^ cytosolic rates or peak amplitude) were calculated by averaging either rates of fluorescence Ca^2+^ change or the amplitude of the fluorescence Ca^2+^ response of number of experiments, n, indicated at the figure legends [16], [32].

The results of the experiments are the mean ± S.E.M. (standard error of the mean) of at least 3 independent experiments (n), using 20–30 cells in each. Statistical significance for all experiments was determined using a one-way ANOVA test followed by Bonfferoni post-hoc analysis. * Significantly different (P<0.05) compared to control.

## Results

### The role of NCLX in mitochondrial Ca^2+^ transport in β cells

**Figure 1:**
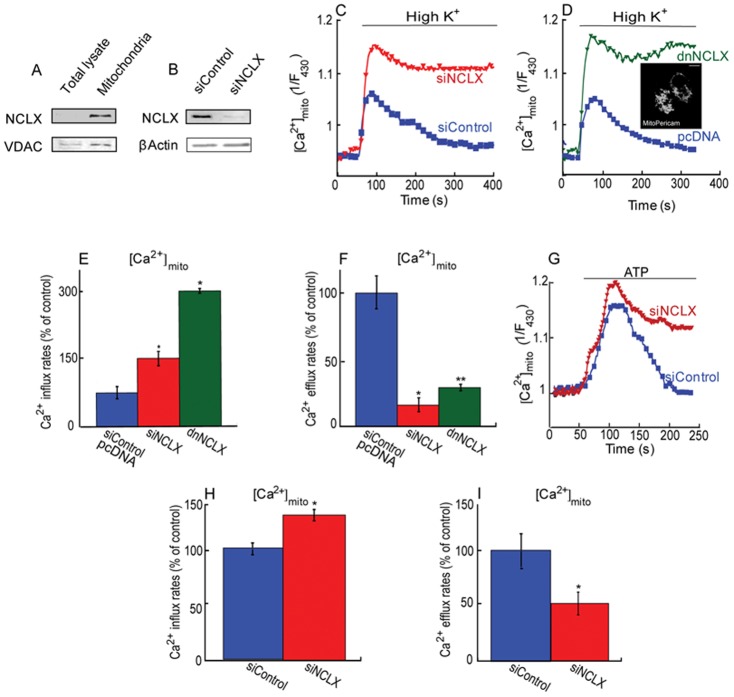
NCLX is expressed in mitochondria of pancreatic β cells and mediates mitochondrial Ca^2+^ transport. **A**. Immunoblot analysis of NCLX expression in total lysate and isolated mitochondria in MIN6 cells (20 µg). **B.** Immunoblot analysis of NCLX expression in siNCLX vs. siControl (20 µg) transfected MIN6 cell lysates. VDAC and β Actin were used as mitochondrial and cytosolic markers, respectively. **C.** Knock down of NCLX expression increases Ca^2+^ influx and inhibits mitochondrial Ca^2+^ efflux. At the indicated time, cells were superfused with high K^+^ Ringer solution while monitoring mitochondrial Ca^2+^ in MIN6 cells transfected with mito-pericam and either siNCLX or siControl. **D.** Dominant negative NCLX construct increases Ca^2+^ influx and inhibits mitochondrial Ca^2+^ efflux. Representative fluorescent traces of pancreatic MIN6 cells co-transfected with mito-pericam and either dnNCLX or control vector (pcDNA), while applying the same experimental paradigm described in Fig. 1C. **Insert.** Representative images of MIN6 cells co-transfected with mito-pericam. The scale bar is 10 µm. **E.** Averaged rates of mitochondrial Ca^2+^ influx of Fig. 1C, D, n = 9 (*P<0.05). **F.** Averaged rates of mitochondrial Ca^2+^ efflux of Fig. 1C, D, n = 9 (*P<0.05). **G.** Silencing of NCLX expression inhibits mitochondrial Ca^2+^ efflux following a metabotropic cytosolic Ca^2+^ response. Cells were co-transfected with mito-pericam and either siNCLX or siControl and superfused with Ca^2+^ free Ringer solution containing 50 µM ATP, while monitoring the Ca^2+^ response. **H.** Averaged rates of mitochondrial Ca^2+^ influx of Fig. 1G, n = 7 (*P<0.05). **I.** Averaged rates of mitochondrial Ca^2+^ efflux of Fig. 1G, n = 7 (*P<0.05).

We first asked if NCLX is expressed in β cells and if it is participating in mitochondrial Ca^2+^ transport. Immunoblot analysis of NCLX in total lysates and isolated mitochondria from MIN6 cells demonstrated that NCLX was enriched in mitochondrial fractions (Fig. 1A), a finding consistent with its primary localization in mitochondria [16]. To further determine if the immunoblot signal is related to NCLX, MIN6 cells were transfected with siNCLX vs. siControl constructs (Fig. 1B). Transfection with siNCLX was followed by a marked decrease in NCLX expression suggesting the ∼50 KDa polypeptide is related to NCLX [16] and indicating that NCLX expression can be molecular controlled.

To determine whether NCLX mediates mitochondrial Ca^2+^ efflux or affecting influx during trans-mitochondrial Ca^2+^ transport, mitochondrial Ca^2+^ transport was determined in MIN6 cells co-transfected with the mitochondrial targeted Ca^2+^ sensor mito-pericam and with either siNCLX or siControl. Following depolarization of the cells with a high K^+^ (50 mM) Ringer's solution, mitochondrial Ca^2+^ influx was observed in the siControl cells and was followed by a robust efflux phase. In contrast, in cells transfected with siNCLX the mitochondrial Ca^2+^ influx rate increased by 50±11% and a dramatic 80±13% reduction of mitochondrial Ca^2+^ efflux was measured (Fig. 1C, E, F). To determine if this effect is directly linked to NCLX activity, we compared mitochondrial Ca^2+^ transport in cells co-transfected with mito-pericam and either the dominant negative dnNCLX or a control vector pcDNA. We again monitored an increase of mitochondrial Ca^2+^ influx rate (200±5%) and a decrease in efflux (65±4%) in the cells transfected with the dnNCLX construct (Fig. 1D, E, F).

In an additional set of experiments, we monitored the mitochondrial Ca^2+^ response following a metabotropic stimulus triggered by ATP in cells superfused with Ca^2+^-free Ringer solution (Fig. 1G). The mitochondrial Ca^2+^ influx rate following of ATP increased by 40±5% and the efflux was reduced by 50±7% during this metabotropic response (Fig. 1H, I).

Altogether, the results of this set of experiments indicate that NCLX is located in the mitochondria of β cells and participates in trans-mitochondrial Ca^2+^ shuttling by affecting primarily the Ca^2+^ efflux but notably also the influx phase following cellular Ca^2+^ influx or release from ER/Ca^2+^ stores.

**Figure 2:**
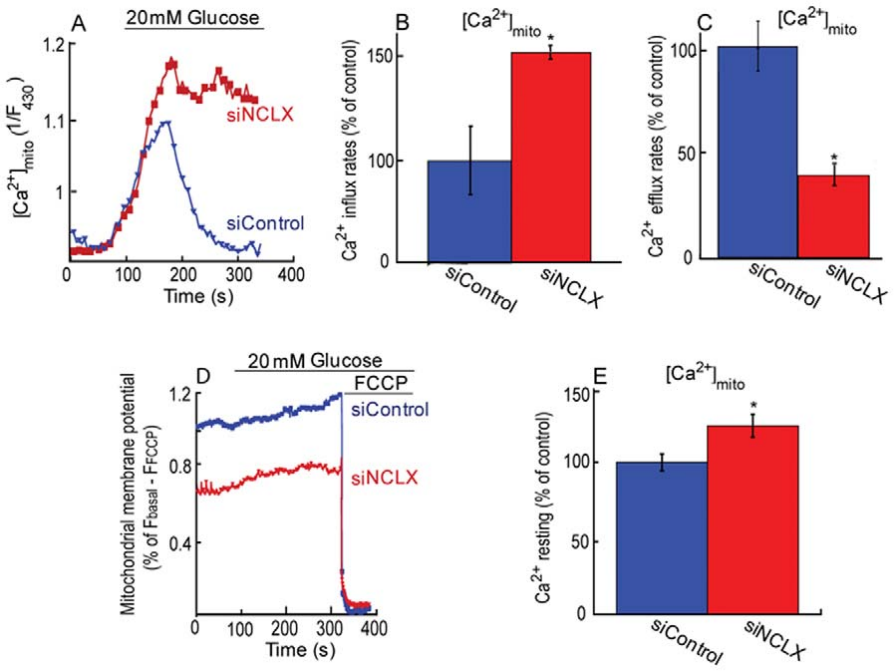
NCLX mediates glucose dependent mitochondrial Ca^2+^ transport and modulates the basal mitochondrial membrane potential and calcium resting levels. **A.** Silencing of NCLX expression blocks the glucose dependent mitochondrial Ca^2+^ efflux. The mitochondrial Ca^2+^ transport was monitored in MIN6 cells co-transfected with mito-pericam and either siNCLX or siControl. Cells were first superfused with low glucose (3 mM) Ringer followed by high glucose (20 mM) Ringer solution. **B.** Averaged rates of mitochondrial Ca^2+^ influx of Fig. 2A, n = 11 (*P<0.05). **C.** Averaged rates of mitochondrial Ca^2+^ efflux of Fig. 2A, n = 11 (*P<0.05). **D.** Silencing NCLX modulates the basal but not glucose dependent change in mitochondrial membrane potential. Changes in mitochondrial membrane potential were monitored in MIN6 cells transfected with siNCLX or siControl, superfused continuously with 0.05 µM TMRM. FCCP 5 µM was added in the indicated times to calibrate the signal. **E.** Effect of knock down of NCLX expression on mitochondrial resting Ca^2+^ in MIN6 cells transfected with siNCLX vs. siControl. Averaged mitochondrial Ca^2+^ basal signals, n = 10 (*P<0.05).

Considering the major effect of NCLX on mitochondrial Ca^2+^ transients, we next sought to determine the role of NCLX on mitochondrial Ca^2+^ responses triggered by high glucose. We further asked if NCLX regulates the mitochondrial membrane potential and resting Ca^2+^ level. Mitochondrial Ca^2+^ was monitored in MIN6 cells that were initially superfused with low glucose (3 mM) followed by application of high glucose (20 mM) Ringer solution. Application of high glucose triggered a robust mitochondrial Ca^2+^ transient (Fig. 2A). Knockdown of NCLX expression was followed by an increase in Ca^2+^ influx (50±4%) and a decrease of Ca^2+^ efflux (56±7%) (Fig. 2B, C). To determine the effect of NCLX on mitochondrial membrane potential, we applied the same experimental paradigm using cells loaded with the mitochondrial membrane potential dye TMRM (0.05 µM). Addition of high glucose was followed consistently with previous studies [33] by small hyperpolarisation of the mitochondria that was unaffected by silencing of NCLX (Fig. 2D). Comparison of the resting mitochondrial membrane potential in siNCLX vs. siControl was performed by comparing the mitochondrial membrane potential before and after the application of FCCP. This analysis indicated however that knocking down NCLX expression led to a small and tonic mitochondrial depolarization (Fig. 2D). We next asked if expression of NCLX is linked to mitochondrial resting Ca^2+^ concentration by comparing basal mito-pericam fluorescence values of siControl vs. siNCLX transfected cells [16]. As shown in Fig. 2E, knockdown of NCLX expression was followed by a minor, but significant increase in resting mitochondrial Ca^2+^ levels. No difference in overall number and cells stained by Trypan Blue was observed between siNCLX vs. siControl indicating that NCLX does not affect the viability of β cells (results not shown).

Altogether, the results of this part indicate that NCLX participates in glucose-dependent mitochondrial Ca^2+^ efflux and also regulates the rate of Ca^2+^ influx transient. Notably, we find that NCLX controls mitochondrial properties, also under resting conditions as the knockdown of NCLX is followed by a mild mitochondrial depolarization and a rise in resting mitochondrial Ca^2+^.

### The role of mitochondrial NCLX in cytosolic calcium changes

**Figure 3:**
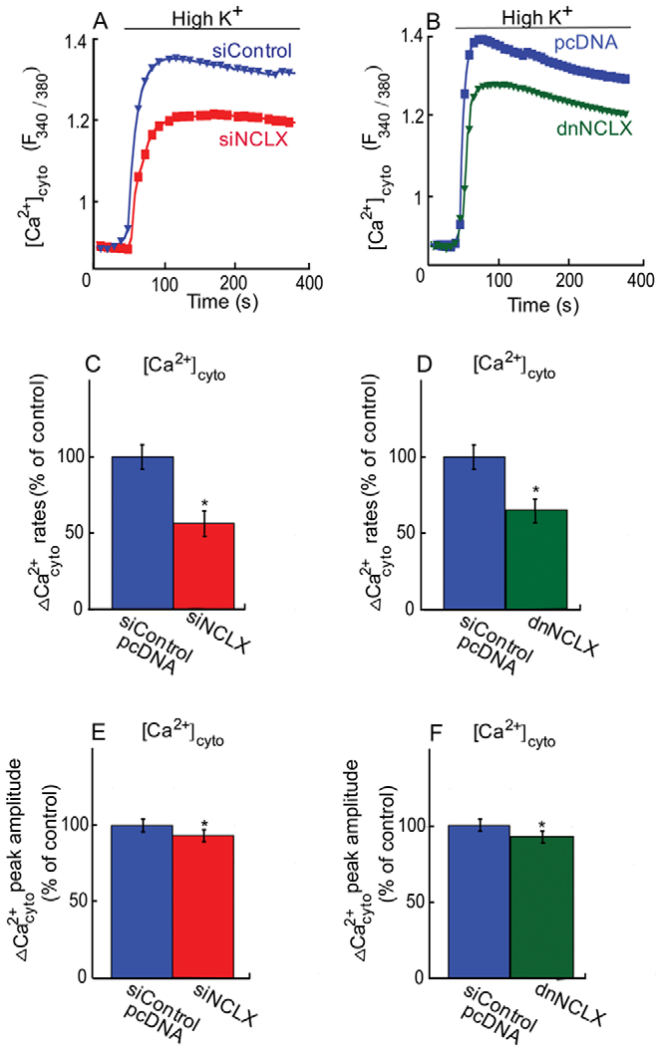
Role of NCLX in cytosolic Ca^2+^ responses. **A.** Silencing of NCLX expression inhibits cytosolic Ca^2+^ responses. MIN6 cells either transfected with siNCLX or siControl were loaded with Fura 2 AM and depolarized with high K^+^ Ringer solution, while monitoring cytosolic Ca^2+^ responses. **B.** Dominant negative mutant NCLX, dnNCLX inhibits cytosolic Ca^2+^ responses. MIN6 cells transfected with either dnNCLX or control vector (pcDNA) were loaded with Fura 2 AM and cytosolic Ca^2+^ was monitored as described in Fig. 3A. **C.** Averaged rates of cytosolic Ca^2+^ responses of Fig. 3A, n = 12 (*P<0.05). **D.** Averaged rates of cytosolic Ca^2+^ responses of Fig. 3B, n = 12 (*P<0.05). **E.** Averaged cytosolic Ca^2+^ response amplitude of Fig. 3A, n = 12 (*P<0.05). **F.** Averaged cytosolic Ca^2+^ response amplitude of Fig. 3B, n = 12 (*P<0.05).

Ionotropic Ca^2+^ influx is a major step in initiating the secretory process in β cells. We have therefore sought to determine the role of NCLX in regulating this process. Cytosolic Ca^2+^ entry was initially triggered in pancreatic MIN6 cells by depolarization with high K^+^ (50 mM) Ringer while monitoring Fura 2 AM fluorescence. We then asked if knockdown of NCLX expression will modulate cytosolic Ca^2+^ signals. Comparison of cytosolic Ca^2+^ rise of siNCLX vs. siControl transfected MIN6 cells showed a marked decrease in the rate of cytosolic Ca^2+^ changes of 45±10% and a decrease of 10±5% in the amplitude of the cytosolic Ca^2+^ signals transfected with siNCLX (Fig. 3A, C, E). We then asked if reducing NCLX activity will also modulate the cytosolic Ca^2+^ signals. The same paradigm was applied to MIN6 cells transfected with either the dominant negative construct of NCLX, dnNCLX, or pcDNA (Fig. 3B) [21]. Similarly to the knocking down of NCLX expression, dnNCLX decreased the rate of cytosolic Ca^2+^ changes and amplitude by 48±10% and 10±5% respectively (Fig. 3B, D, F).

Thus, the results of this set of experiments indicate that NCLX activity plays a major role in controlling both the rate and amplitude of cytosolic Ca^2+^ responses.

### The role of mitochondrial NCLX in glucose-induced cytosolic calcium signals

The above results identified NCLX as the mitochondrial exchanger in β cells and highlight its role in mediating the cross talk with plasma membrane Ca^2+^ influx pathways. The physiological stimulus linked to insulin secretion is however mediated by glucose. Further although MIN6 are a well-established model for β cells, the primary β cells manifest a clearer link between glucose, Ca^2+^ signalling and insulin secretion [34]. We therefore next sought to determine the role of NCLX in shaping cytosolic Ca^2+^ transients triggered by glucose by controlling the expression of NCLX using siNCLX in primary β cells.

**Figure 4:**
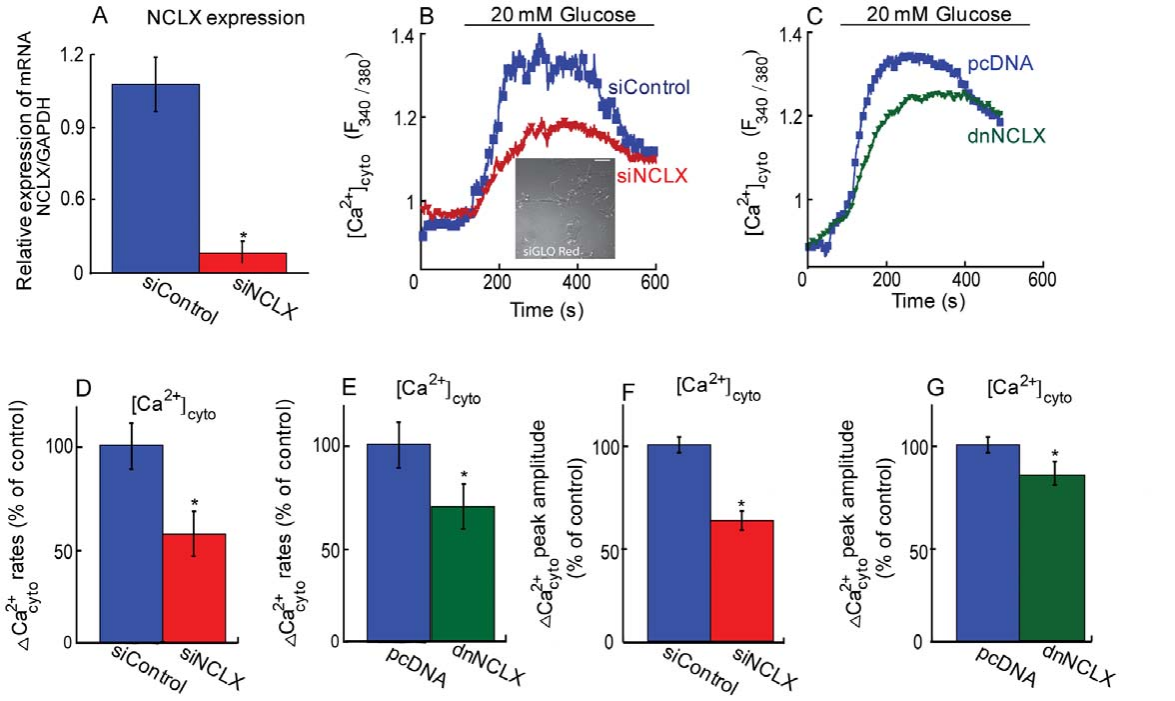
Effect of NCLX expression or activity on glucose dependent cytosolic calcium responses. **A.** Real time PCR analysis of mRNA NCLX expression normalized to GAPDH in pancreatic primary β cells transfected with siNCLX vs. siControl, n = 3 (*P<0.05). **B.** Silencing NCLX expression inhibits glucose-induced Ca^2+^ entry in primary β cells. Representative fluorescent traces of cytosolic Ca^2+^ in pancreatic primary β cells transfected with either siNCLX or siControl loaded with Fura 2 AM and stimulated with high glucose following the same experimental paradigm described in Fig. 2A. **Insert.** Shows representative images of MIN6 cells co-transfected with the Dharmacon siGLO Red transfection reagent. The scale bar is 10 µm. **C.** NCLX dominant negative construct inhibits glucose dependent cytosolic Ca^2+^ changes in primary β cells. Representative fluorescent traces of primary β cells transfected with dnNCLX or control vector (pcDNA) loaded with Fura 2 AM and treated with high glucose when indicated. **D.** Averaged rates of cytosolic Ca^2+^ responses of Fig. 4B, n = 10 (*P<0.05). **E.** Averaged rates of cytosolic Ca^2+^ responses of Fig. 4C, n = 10 (*P<0.05). **F.** Averaged cytosolic Ca^2+^ amplitudes of Fig. 4B, n = 10 (*P<0.05). **G.** Averaged cytosolic Ca^2+^ amplitudes of Fig. 4C, n = 10 (*P<0.05).

Efficacy of siNCLX delivery in primary β cells was determined by co-transfection with siGlo fluorescent marker (see *Methods*), which indicated that the siGLO constructs were taken up by at least 80% of the cells. Consistent with this high transfection rate, efficacy of NCLX knockdowns determined by real-time PCR analysis (Fig. 4A), showed at least a 10 fold reduction in mRNA NCLX in cells transfected with siNCLX. As expected, application of high glucose triggered an increase in the cytosolic Ca^2+^ response in control cells (Fig. 4B). Remarkably, a 43±15% (Fig. 4B, D, F) reduction in the rate of cytosolic Ca^2+^ changes as well as a decrease of 40±5% of the amplitude of Ca^2+^ signals in cells transfected with the siNCLX was observed, indicating the importance of the mitochondrial exchanger, NCLX, for enhancing the rise of glucose-dependent cytosolic Ca^2+^. The knock down of NCLX activity using dnNCLX (Fig. 4C) led to a similar reduction both in the rates of cytosolic Ca^2+^ changes by 35±17%, and in the amplitude of Ca^2+^ signals by 30±8% (Fig. 4C, E, G).

Hence, this set of experiments indicates that NCLX shapes the rate and amplitude of glucose-dependent cytosolic Ca^2+^ responses in primary β cells.

### The role of mitochondrial NCLX in glucose dependent mitochondrial calcium homeostasis

**Figure 5:**
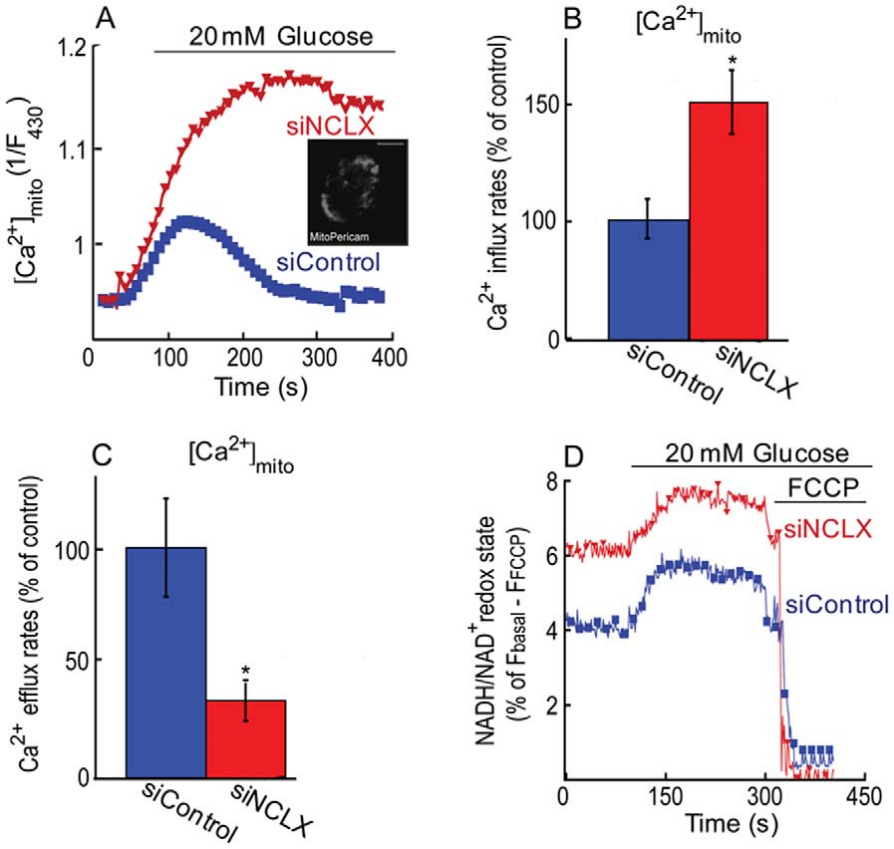
Effect of NCLX on mitochondrial Ca^2+^ transport, metabolic rate in resting and high glucose dependent manner. **A.** Knocked down of NCLX modulates mitochondrial calcium transport. Pancreatic primary β cells were infected with lenti-pericam viral particles and transfected with either siNCLX or siControl and superfused with the indicated high glucose Ringer solution. **Insert.** Representative image of pancreatic primary β cell infected with lenti-pericam. The scale bar is 10 µm. **B.** Averaged mitochondrial Ca^2+^ influx rates of pancreatic primary β cells of Fig. 5A, n = 3 (*P<0.05). **C.** Averaged mitochondrial Ca^2+^ efflux rates of Fig. 5A, n = 3 (*P<0.05). **D.** Effect of NCLX on respiratory chain activity determined by monitoring NAD(P)H intrinsic fluorescence in pancreatic primary β cells, transfected with either siNCLX or siControl before and after application of high glucose Ringer solution. FCCP or high glucose Ringer's solution was added where indicated.

We next sought to determine if NCLX determines the trans-mitochondrial Ca^2+^ transient and metabolic rate manifested by the ratio of NAD(P)H/NAD^+^. The mitochondrial Ca^2+^ transient signal was monitored in primary β cells co-transfected with a lenti viral construct of mito-pericam (Fig. 5A), following the same experimental procedure described in Fig. 4B. The mitochondrial rate of Ca^2+^ uptake was increased by 50±15% and the Ca^2+^ efflux rates were strongly reduced by 70±7% (Fig. 5B, C) in pancreatic primary β cells transfected with siNCLX compared to the rates measured in siControl transfected cells.

We then determined the effect of NCLX expression on the ratio of NAD(P)H/NAD^+^ by measuring NADH autofluorescence. Consistent with previous studies [35], addition of high glucose Ringer's solution was followed by enhanced NAD(P)H production. Silencing NCLX expression did not affect the stimulatory effect of high glucose on NAD(P)H (Fig. 5D). However, determination of NAD(P)H/NAD^+^ fluorescence following addition of the FCCP indicated that the basal metabolic rate in cells transfected with siNCLX was enhanced (Fig. 5D) suggesting that the slightly elevated resting mitochondrial Ca^2+^ triggered by the knock down of NCLX expression (Fig. 5A) was sufficient to enhance basal NAD(P)H/NAD^+^ ratio.

Altogether, the results of this part suggest that NCLX is regulating the rates and amplitude of influx and efflux phases of mitochondrial Ca^2+^ transients induced by high glucose and it is also affecting the basal NAD(P)H production rates.

### The link between NCLX, glucose-dependent ATP production and insulin secretion

**Figure 6:**
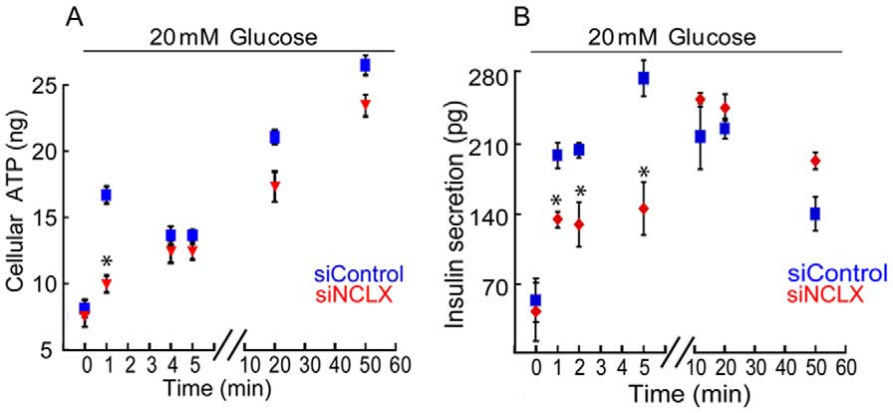
Effect of NCLX silencing expression on ATP production and insulin secretion. **A.** Effect of NCLX silencing expression on ATP production. The ATP content was determined in pancreatic primary β cells lysates transfected with either siNCLX or siControl and stimulated with high glucose in the indicated times (*see Experimental Procedures*), n = 3 (*P<0.05). **B.** Effect of NCLX knocked down expression on glucose dependent insulin secretion. Cultured pancreatic primary β cells were transfected with either siNCLX or siControl and amounts of secreted insulin were determined in the indicated times, n = 3 (*P<0.05).

We next determined the time course of glucose-dependent ATP production by monitoring luciferase luminescence in primary β cells lysates [13]. In general, rate of glucose-dependent ATP production was not affected by NCLX expression; it was however transiently reduced at 1 min by 37±0.5% following glucose application in the NCLX knock down cells compared to control (Fig. 6A).

Finally, considering the major role of NCLX on glucose-dependent cytosolic Ca^2+^ response, we asked if it also participates in regulating the rate of insulin secretion. We used primary β cells transfected with siNCLX vs. siControl while monitoring insulin secretion before and after application of high glucose Ringer's solution. The supernatant of treated cells was collected at the indicated time intervals and amounts of secreted insulin levels were determined. Partial inhibition by 40±8% - 1 min, 40±14% - 2 min, 60±16% – 5 min, in the rate of insulin secretion was monitored in siNCLX primary β cells during the first 5 minutes (Fig. 6B). This trend was however reversed and amounts of secreted insulin were similar at latter time points following application of glucose in siNCLX vs. siControl treated primary β cells.

Thus, our results indicate that NCLX has a small effect on high glucose dependent ATP production, but primarily regulates rates of glucose-dependent insulin secretion particularly during the first phase of insulin secretion.

## Discussion

The activity of the mitochondrial Na^+^/Ca^2+^ exchanger and its role in Ca^2+^ signalling leading to insulin secretion is of major interest because of the dual role of mitochondrial Ca^2+^ shuttling on mitochondrial metabolism and on global Ca^2+^ signalling [36]. The activity of major players in mitochondrial Ca^2+^ shuttling, the uniporter and exchanger in pancreatic β cells, has been documented in several studies [37], [38]. However, their role in regulating Ca^2+^ signals, and subsequent insulin secretion remains controversial because it was unclear whether their inhibitors might modulate non-specifically other Ca^2+^ transporters [14], [15], thus underscoring the importance of a molecular based approach to analyse the role of mitochondria in these processes.

Towards this goal, we first asked if NCLX, recently identified by our group as a candidate for the mitochondrial Na^+^/Ca^2+^ exchanger [16], plays a similar role in pancreatic β cells, and if molecular tools aimed at inhibiting its expression or activity can be used to analyse Ca^2+^ signalling and secretion in these cells. Our results indicate that NCLX is the mitochondrial exchanger in pancreatic β cells and that its expression or activity can be molecularly targeted based on the following findings: 1) NCLX is localized in the mitochondria of β cells, 2) silencing of NCLX expression leads to inhibition of mitochondrial Ca^2+^ efflux, 3) transfection of β cells with a dominant negative NCLX construct has a similar inhibitory effect on mitochondrial Ca^2+^ efflux activity, 4) both knock down of NCLX expression or activity inhibit mitochondrial calcium efflux following a glucose-dependent Ca^2+^ rise. However, our results indicate that NCLX in β cells has an additional role that has not been documented in previous studies [16]. We show that silencing of NCLX expression or activity also enhances the rate of mitochondrial Ca^2+^ influx in β cells. This suggests that the Ca^2+^ efflux mediated by NCLX is strongly activated already at the early phase of mitochondrial Ca^2+^ influx and thus, NCLX has the capacity to shape not only the mitochondrial Ca^2+^ efflux phase but also indirectly the Ca^2+^ rise in this organelle. This effect is particularly remarkable considering that the influx phase, that was previously monitored in many cell types is about 2 orders of magnitude faster than the efflux phase [39]. Consistent with this dominant role of NCLX on the mitochondrial Ca^2+^ response, we observed that NCLX determines the resting levels of mitochondrial Ca^2+^ in β cells, a finding that may explain the role of NCLX in metabolic processes (discussed below). These major effects of NCLX are consistent with the high pancreatic expression of NCLX in β cells [31].

In our recent report [44] describing the role of the mitochondrial uniporter MCU [5], [4] in single β cells, preliminary observations suggested that silencing of NCLX modulated the amplitude of mitochondrial calcium increase in response to stimulated Ca^2+^ influx. The effects of NCLX elimination on mitochondrial calcium changes prompted by physiological stimulation with glucose were not explored, nor were the kinetics of mitochondrial transport, mitochondrial membrane potential or glucose-regulated insulin secretion. In the detailed studies described here we now show that NCLX plays a key role in regulating the glucose-dependent Ca^2+^ response in the cytosol and the mitochondria. Our results indicate that NCLX activity is critical for clearance of mitochondrial Ca^2+^ and is therefore a rate limiting player in the mitochondrial Ca^2+^ response induced by glucose. In addition, we demonstrated that by catalysing the mitochondrial efflux, NCLX also shapes the cytosolic glucose-dependent Ca^2+^ response and thereby, regulates the rate of insulin secretion.

Mitochondria are occupying vastly different relative volumes in different cell types and play a highly heterologous role in regulating cytosolic Ca^2+^ in distinct tissues. For example, in cardiac tissue they occupy ∼30% of the total volume [40], but play a relatively minor role in shaping the cytosolic Ca^2+^ responses [41]. In contrast, in chromaffin cells [10] the estimated mitochondrial cell occupancy is only about 6%, yet the mitochondria play a major role in cytosolic Ca^2+^ uptake. The estimated occupancy of mitochondria in β cells is even lower at about 4% [42], however our findings indicate that despite their relatively modest volume, they are playing a major role in shaping the Ca^2+^ signalling of β cells. We find that silencing either the expression or the activity of NCLX, decreases the rate of cytosolic Ca^2+^ changes by glucose by approx. 40% and the amplitude of the Ca^2+^ signals by 30%. Considering the small volume occupied by the mitochondria and the large change that it triggers in cytosolic Ca^2+^, our results indicate that it outpaces by several fold, the transport rate mediated by the plasma membrane and ER Ca^2+^ transporters. Remarkably, despite the major cytosolic Ca^2+^ changes triggered by NCLX, the mitochondrial changes are relatively modest. Several studies have underscored the powerful Ca^2+^ buffering capacity of mitochondria, in particular the formation of calcium phosphate, that is at least 10 fold stronger than the buffering capacity of the cytosolic Ca^2+^ [43]. Furthermore, mitochondrial Ca^2+^ buffering, dependent on matrix pH, allows a rapid dissociation of Ca^2+^ phosphate, thus readily providing the exchanger with Ca^2+^ [44]. Such mechanism can explain how the small changes in mitochondrial Ca^2+^ provide sufficient amounts of Ca^2+^ required to change its cytosolic concentrations.

Recent studies have highlighted the physical proximity and functional cross-talk between the L-type calcium channels, LTCC and the mitochondrial network. This leads to a robust LTCC-dependent rise of local Ca^2+^ in the vicinity of mitochondria followed by enhanced mitochondrial Ca^2+^ uptake [45]. A rapid uptake by mitochondria may provide a fast Ca^2+^ clearing mechanism thereby minimizing the potential toxic effect of a strong cytosolic Ca^2+^ surge. On the other hand, a rapid decline in cytosolic Ca^2+^ may interfere with the first phase of insulin secretion, which requires a sufficient rise in cytosolic Ca^2+^ because of the low Ca^2+^ affinity of the secretory machinery [46]. Previous studies have indeed underscored the requirements for sustained Ca^2+^ rise during the first phase of insulin secretion [47]. In contrast, the second phase is dominated by the R-Type Ca^2+^ channels and requires a more moderate Ca^2+^ rise [47], [48]. Our results suggest that NCLX is tuning the cytosolic Ca^2+^ levels in β cells by mediating a continuous and robust mitochondrial Ca^2+^ efflux, thereby preventing rapid cytosolic Ca^2+^ decline and augmenting sustained elevated cytosolic Ca^2+^ levels required for this secretory phase. This role in secretion is supported by our finding demonstrating a delay in glucose-dependent insulin secretion triggered by silencing of NCLX. Thus, we suggest a more dominant role for strong Ca^2+^ rise during the first phase of insulin secretion. An important finding of this study is that the role of the mitochondrial exchanger NCLX in regulating the cytosolic Ca^2+^ determines the rate of insulin secretion during the initial phase of secretion. Previous studies using the mitochondrial exchanger inhibitor CGP-37157 suggested that the inhibition of the exchanger led to enhanced ATP production and insulin secretion [12]. In contrast, and consistent with similar observations [49], [13] we find that knock down of NCLX expression does not lead to enhanced glucose-dependent ATP production. This effect may be related to the role of NCLX in shaping the basal mitochondrial resting Ca^2+^ in β cells. Our results indicate that the silencing of NCLX leads to a small but significant rise in basal mitochondrial resting Ca^2+^. Because the affinity of the Ca^2+^ sensitive Kreb's cycle enzymes is relatively high, even such a modest rise in Ca^2+^ may be already sufficient to induce their activation at low levels of glucose [50]. Indeed, the metabolic pathway is highly sensitive to even small changes in mitochondrial Ca^2+^. Consistent with such a mechanism, we observed that the acceleration of the basal metabolic rate was induced following knockdown of NCLX expression. In addition, knockdown of NCLX is followed by partial mitochondrial depolarization that may further decrease the efficiency of ATP production. Alternatively, the rise in resting mitochondrial Ca^2+^ could trigger the formation of oxygen radicals of NO species that may have an inhibitory effect on metabolic processes in β cells [51]. Therefore, an important conclusion of this study is that effects on resting metabolic rate triggered by inactivation of NCLX are likely to play a mitigating role in the potentially stimulatory effect of NCLX knockdown on ATP production during the high glucose phase. Thus, our findings argue against a major energetic role played by the exchanger in Ca^2+^ signalling linked to insulin secretion as previously suggested based on the inhibition of the exchanger by CGP-37157. The latter effect may have also been related to the modulatory effect of this compound on other major Ca^2+^ pathways such as the L-type Ca^2+^ channel or SERCA, that can affect ATP consumption and thereby could indirectly change the energy balance in β cells [52]. Instead, our results indicate that the Ca^2+^ transport activity mediated by NCLX and its strong effect on increasing cytosolic Ca^2+^ responses, are the primary roles of NCLX that are linked to insulin secretion. Further *in vivo* studies, employing for example transgenic NCLX knockout mice, will be required to study the contribution of mitochondrial Ca^2+^ shuttling on these aspects of islet physiology.
